# Individual Characteristics and National Income Modify the Association between Cognitive Social Capital and Food Insecurity: Evidence from the Gallup World Poll, 2014–2021

**DOI:** 10.1016/j.cdnut.2026.107654

**Published:** 2026-02-06

**Authors:** Sejla Isanovic, Kegan O’Connor, Audrey L Richards, Edward A Frongillo

**Affiliations:** 1 Department of Health Promotion, Education, and Behavior, Arnold School of Public Health, University of South Carolina, Columbia, SC, United States; 2 United States Department of Agriculture, Economic Research Service, Washington, DC, United States

**Keywords:** food insecurity, social capital, Gallup World Poll, stress-support theory, multilevel analysis

## Abstract

**Background:**

Food insecurity affects >2.4 billion people globally and persists across income levels. Characteristics such as low income, low education, and unstable employment do not fully explain this persistence. Social resources embedded in networks, specifically cognitive social capital involving trust, reciprocity, and support, may offset constraints and buffer characteristics associated with a higher probability of food insecurity.

**Objectives:**

This study examined whether social capital was associated with a lower probability of food insecurity and whether this association varied by individual characteristics and country contexts, consistent with buffering and compensation.

**Methods:**

Data were drawn from the Gallup World Poll (2014–2021), comprising 702,850 respondents aged ≥15 y across 115 countries. Moderate or severe food insecurity was assessed using the 8-item Food Insecurity Experience Scale; social capital was measured using a binary indicator. Six individual-level characteristics were tested using multilevel linear probability models with country fixed effects and interaction terms. Country-specific slope coefficients capturing the association between social capital and food insecurity, obtained from a random-coefficient model, were regressed on log-transformed gross national income (GNI) per capita.

**Results:**

The mean probability of food insecurity was 0.259; the mean prevalence of social capital was 0.820. In all countries, higher social capital was associated with a lower probability of food insecurity (slopes −0.318 to −0.055); quadratic analysis of the slopes on log-transformed GNI per capita showed consistent slopes in low- and middle-income countries and steeper slopes in high-income countries (*P* < 0.001). Among individuals, associations were largest with primary education (−16.96 pp), low income, unemployment (−20.4 pp), poor health (−18.44 pp), and widowhood (−19.68 pp).

**Conclusions:**

The strength of the negative association between social capital and food insecurity varied by individual characteristics and national income, consistent with buffering and compensation.

## Introduction

Food insecurity remains a pressing global concern, affecting people across all income levels. Food insecurity occurs when a person lacks access to safe and nutritious food, and can impact growth, development, and health [1]. In 2022, ∼2.4 billion people experienced moderate to severe food insecurity worldwide [2]. Food insecurity is not confined to low-income settings: analysis of data from 34 high-income countries between 2014 and 2018 indicated that 6.5% of adults experienced moderate or severe food insecurity, and 1.6% experienced the most severe levels [3]. Well-established determinants such as low income, low educational attainment, and unstable employment are strong predictors of food insecurity but do not fully account for the persistence of food insecurity, even in contexts where assistance programs exist [4].

Educational attainment, employment status, income, marital status, physical health, and residency are characteristics that are associated with the experience of food insecurity that could be modified by social capital. Social capital is defined as the networks, norms, and trust that facilitate collective action [5,6]. Social capital has multiple components, including both structural and cognitive. Structural social capital refers to the observable features of social networks and social participation (e.g., network ties and civic engagement) through which social resources can be accessed and used. Structural social capital manifests through bonding ties (connections with close-knit, homogeneous groups), bridging ties (connections across diverse groups), and linking ties (connections through vertical links to institutions) [4,7]. Cognitive social capital refers to the perceived quality and content of social relationships, encompassing trust, reciprocity, and mutual support [8,9].

**FIGURE 1:**
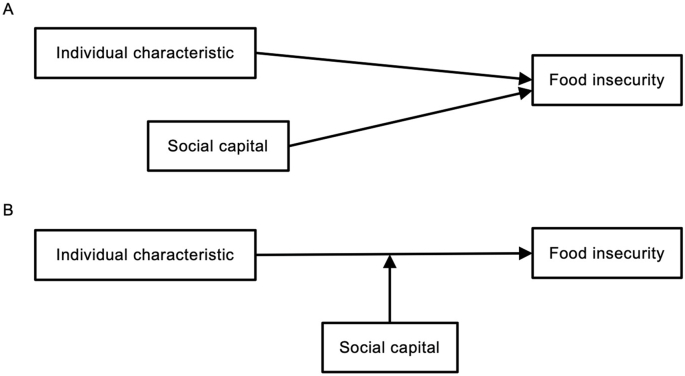
Conceptual framework illustrating compensation and buffering linking cognitive social capital and food insecurity. (A) Compensation in which cognitive social capital is directly associated with a lower probability of food insecurity, independent of an individual characteristic associated with a higher probability of food insecurity. (B) Buffering in which the association between food insecurity and an individual characteristic associated with a higher probability of food insecurity is attenuated by higher cognitive social capital.

From the perspective of the life stress model [10,11], social capital operates in 2 ways. Within the life stress model, external stressors (e.g., role strains and economic shocks) increase risk of adverse outcomes, whereas psychosocial resources influence outcomes by operating through *1*) a compensatory process that lowers risk irrespective of stress exposure and *2*) a buffering process that weakens the stress–outcome association by shaping appraisal, coping, and behavioral processes. In this conceptualization, social capital, composed of networks, trust, and reciprocity, functions as a psychosocial resource that can show both compensation with lower food insecurity and buffering that attenuates the association of individual characteristics associated with a higher probability of food insecurity. In this study, compensatory denotes a direct association of social capital with a lower probability of food insecurity that does not differ across individual strata. Accordingly, if income and other individual characteristics are not found to modify the relationship between social capital and food insecurity, we interpret the effect as compensatory (Figure 1A); if the association is stronger in magnitude among individual characteristics associated with a higher probability of food insecurity, we interpret it as buffering (Figure 1B).

In a global sample of 134 countries, Smith et al. [12] reported a consistent association between cognitive social capital, measured as having relatives or friends to count on for help, and food insecurity across World Bank economic development ranks, suggesting compensation rather than modification. Within the United States, regional differences were associated with lower food insecurity; bonding capital was dominant in southeast regions, and bridging and linking capital were more prominent in northwest regions [4]. In Turkey, linking social capital, operationalized as political ties and engagement with the ruling party, was associated with respondents’ perceived improvement in economic well-being over the prior 5 y, with patterns differing between rural and urban contexts [13]. In rural Malawi, greater social capital (e.g., participation in farmers’ organizations, larger social networks, and voluntary group engagement) was positively associated with improved household food security. Dzanja et al. [7] found that when incorporating social capital into their models, the explanatory power improved by ∼15%. Households with larger social networks in Northern Uganda were found to be twice as likely to be only moderately (compared with severely) food insecure when compared with households with smaller networks [14]. In periurban Peru, stronger community-level social connectedness predicted higher household food security even after adjusting for socioeconomic status [15].

Given this context-specific variability, it remains unclear under which conditions social capital shows compensation or buffering in relation to food insecurity. Smith et al. [12] emphasized that social capital was associated with lower food insecurity across economic contexts but did not investigate the variability or specific conditions influencing this relationship. Distinguishing whether social capital shows a compensatory association with food insecurity or modifies (i.e., buffers) the association between individual characteristics and food insecurity clarifies design targets for causal studies and evaluation. This distinction operationalizes the stress model into testable hypotheses: compensation would predict a lower mean probability of food insecurity where social resources are more prevalent, whereas buffering would predict stronger associations among individuals facing specific stressors [10,11]. Previous studies have typically used single-country analyses or multicountry designs that estimate associations without explicitly testing interactions [4,7,[12], [13], [14], [15]]. This study examines whether and how individual characteristics and country-level conditions influence the relationship between cognitive social capital and food insecurity, specifying the conditions under which cognitive social capital shows compensation or buffering.

## Methods

### Data and sampling

Repeated cross-sectional data for this study were obtained from the Gallup World Poll (GWP) conducted in 8 survey years between 2014 and 2021 [16,17]. The GWP is an annual, nationally representative survey that draws independent samples each year. The sampling frame represents the civilian, noninstitutionalized population aged ≥15 y living in households; individuals in group quarters and other institutional settings are not included. Countries did not contribute data for the same number of survey years; across the 115 countries in the analytic sample, the number of available survey years ranged from 1 to 8. Four countries had data only for the last survey year (2021), 15 countries had data for 3–5 survey years, and the remaining 96 countries had data for 6–8 survey y with 8 survey years as the mode (40 countries). The GWP used multistage sampling designs that differed by country. In telephone surveys, households were selected using random digit dialing or through nationally representative lists of phone numbers; where cell phones predominated, a dual sampling frame was used. In countries where face-to-face interviewing was conducted, sampling occurred in 3 stages: *1*) clusters of households were stratified by population size and geography and selected proportionally, when possible, *2*) households were chosen via a random-route procedure, and *3*) respondents were randomly selected based on the latest birthday or using a Kish grid method. The GWP survey instrument, translated into the major languages of each country, includes a core set of questions on topics such as law and order, food and shelter, institutions and infrastructure, job climate, and various dimensions of well-being. In 2014, the Food Insecurity Experience Scale (FIES) was introduced in the GWP [18].

Responses to the 8 FIES items were summed to obtain a raw score (0–8). To ensure cross-country comparability, the FAO uses a 1-parameter Rasch model to equate each national FIES scale to a global standard scale and to define cut-points for moderate and severe food insecurity [19]. These cut-points are mapped to country-specific raw-score thresholds (typically ≥4 for moderate or severe and ≥7 for severe). Consistent with prior work [12], food insecurity was operationalized as a binary indicator and assigned a value of 1 when a respondent’s raw score met or exceeded the country-specific moderate or severe threshold, 0 otherwise. At the respondent level, the variable is categorical; in regression analyses, its mean and coefficients are interpreted as probabilities. A linear probability model was estimated, and coefficients were reported as percentage-point changes in the probability of experiencing moderate or severe food insecurity. Social capital was based on respondents’ report of whether they have relatives or friends to count on for help. Cognitive social capital was measured using the GWP item: “If you were in trouble, do you have relatives or friends you can count on to help you whenever you need them, or not?” [12,16]. This item aligns with the definition of cognitive social capital, people’s experiences of trust, reciprocity, and mutual support [4]. Response options were “Yes” and “No,” with “Don’t know” and “Refused” recorded when applicable. We coded “Yes” as 1 and “No” as 0. Responses coded as “Don’t know” (*n* = 6260) or “Refused” (*n* = 518) were excluded; analyses used 702,850 respondents with substantive “Yes” (*n* = 576,429) or “No” (*n* = 126,421) responses.

Six individual-level characteristics, expressed as categories as provided by GWP, were selected as potential modifiers for this analysis: employment status, education attained, income, marital status, physical health, and residency. These characteristics capture key resource, human-capital, and social-support dimensions that prior literature has frequently reported as associated with food insecurity [[20], [21], [22], [23], [24]]. These characteristics also provide broad observational coverage in the dataset. Individual-level characteristics with limited data, like education access—which had ∼60,000 observations—were not used to prioritize variables with the most comprehensive data availability (Supplementary Table 1).

Employment status was categorized into 6 mutually exclusive groups based on work hours, employment type, and job-seeking status (employed full-time for an employer; self-employed full-time; employed part-time and do not want to work full-time; employed part-time and want to work full-time; unemployed; and out of the workforce). Individuals “employed full-time for an employer” worked ≤30 h/wk for an employer, and those “employed full-time for self” worked ≤30 h/wk in self-employment. Part-time employment was classified as having worked >30 h/wk. Part-time employment was divided into 2 categories: individuals “employed part-time who do not want full-time work” (i.e., did not wish to increase their hours); and individuals “employed part-time who want full-time work” (i.e., desired to work >30 h/wk). The “unemployed” category consisted of individuals who had not worked in the past 7 days for an employer or themselves. Individuals classified as “out of the workforce” had also not worked in the past 7 d but were not actively seeking a job or were unavailable to start work [16].

Educational attainment was harmonized into 3 categories (primary or less, secondary, and college degree or equivalent). Annual household income was measured in international $ using a 27-bracket ordinal scale based on worldwide income distribution and converted using purchasing power parity (PPP) ratios; this variable was treated as continuous after confirming that alternative specifications did not improve model fit [16]. Marital status was categorized as single/never married, married, living with a domestic partner, separated, divorced, and widowed. Physical health was measured by asking respondents to report whether health problems were preventing them from doing things that people their age normally can do. Residency was categorized into 4 groups: a rural area or on a farm, a small town or village, a large city, and a suburb of a large city [16,18].

We examined whether the association between cognitive social capital and food insecurity varied across strata of individual characteristics that capture differences in socioeconomic resources (income, educational attainment, employment status), health-related functional limitation (physical health limitation), and social and residential context (marital status, residency). These modifiers were specified to represent empirically observed strata that may modify the association between social capital and food insecurity.

For categorical modifiers, indicator variables were created, and 1 category was designated as the reference to facilitate the interpretation of contrasts. Reference categories were selected to provide interpretation across the pooled, multicountry sample. Specifically, we used *1*) employed full time for an employer as the reference category for employment status; *2*) primary education as the lowest educational attainment category; *3*) single/never married as the reference category “not currently partnered” for contrasts with currently partnered statuses (married and domestic partner) and previously partnered statuses (separated, divorced, widowed); *4*) presence of an activity-limiting health problem (yes) as the reference category for the functional limitation indicator; and *5*) rural as the reference category for urbanicity contrasts (small town/village, large city, and suburb).

### Ethics considerations

The analyses used deidentified individual-level data from the GWP (2014–2021), exempting the analyses from ethical review. The data were provided to the authors under a data-use agreement with the USDA, Economic Research Service. Respondents provided consent before the interview.

### Statistical analyses

Analyses were conducted using Stata Statistical Software: Release 17 [25]. Descriptive statistics (means, SDs, and percentages) were calculated to summarize the sample characteristics. The outcome, moderate or severe food insecurity (range: 0–1) derived from the FIES, was modelled with a linear probability model. Linear probability models express associations in percentage-point units, accommodate high-dimensional fixed effects and interaction terms, and perform well with large samples [26,27]. The predictor was social capital. Interaction terms were included to test whether the association between social capital and food insecurity differed across values of (i.e., was modified by) individual-level characteristics. Six fixed-effects regression models were estimated to examine the modification by employment status, educational attainment, income, marital status, physical health, and residency in the relationship between social capital and food insecurity. Fixed-effects models were used to account for unmeasured country-level characteristics and absorb all time-invariant country-level heterogeneity [28,29]. No covariates were included because the focus was on the interactions; any covariate heterogeneity across countries was absorbed. Survey year was included as a fixed effect to account for differences by year of data collection. The sample sizes were driven primarily by the availability of data on social capital. Given the small proportion of missing data for the other variables included in each model, we analyzed complete cases.

A random-coefficient, mixed-effects model was estimated to examine the relationship between social capital and food insecurity while accounting for potential heterogeneity across countries. This model allowed both the intercept (i.e., the baseline probability of food insecurity in the absence of social capital) and the slope for social capital to vary randomly by country. An unstructured covariance matrix was specified to freely estimate the variance and covariance of the random effects, capturing between-country variability.

To investigate whether differences in national income levels help explain variation in the association of social capital with food insecurity, country-specific estimates of the association (i.e., the random slopes) were regressed on mean national income, measured as the natural log of gross national income (GNI) per capita in PPP terms. Data on each country’s GNI per capita (PPP, current international $, World Bank indicator NY.GNP.PCAP.PP.CD) [30] were calculated as the mean for 2014–2021 and transformed with the natural logarithm. Visual inspection using a locally weighted regression plot indicated curvature in the relationship; therefore, a quadratic log-GNI term was included. Higher-order polynomial terms did not improve model fit, i.e., did not increase the R-square or reduce the root mean square error. We used ordinary least squares, which provides unbiased estimates but may not be optimally efficient in the presence of heteroskedasticity [31]. Any potential inefficiency was not a concern because the regression coefficients were estimated with narrow confidence intervals and small *P* values.

## Results

### Sample characteristics

The dataset comprised 824,598 observations from 115 countries contributing data on household and individual socioeconomic conditions (Table 1). Overall, the mean probability of moderate or severe food insecurity was 0.259 (*n =* 824,598, SD = 0.394). Social capital levels were high, averaging 0.820 (*n =* 702,850, SD = 0.384) on a scale from 0 to 1. For employment status, 29.0% of respondents were employed full-time for an employer, 13.8% were self-employed full-time, 7.30% worked part-time (with or without a preference for full-time work), 6.18% were unemployed, and 36.4% were out of the workforce.

**TABLE 1:** Sample characteristics for datasets from the Gallup World Poll 2014–2021, 115 countries

Characteristic	Observations	Mean ± SD/percentage (%)
Food insecurity (moderate or severe)^1^	824,598	0.259 ± 0.394
Social capital (range, 0–1)^2^	702,850	0.820 ± 0.384
Employment status	817,254	—
Employed full-time for an employer	—	29.0
Employed full-time for self	—	13.8
Employed part-time, do not want full-time	—	6.89
Employed part-time, want full-time	—	7.71
Unemployed	—	6.18
Out of workforce	—	36.4
Educational attainment	820,837	—
Primary	—	28.4
Secondary	—	51.7
College	—	19.8
Income (annual international dollars, range, 1–27)^3^	824,497	13.4 ± 6.34
Marital status	816,405	—
Single/never married	—	28.9
Married/domestic partner	—	56.9
Separated/divorced	—	6.67
Widowed	—	7.47
Health problems preventing normal activities (yes)	751,736	24.8
Place of residence	821,862	—
Rural area or farm	—	23.3
Small town or village	—	34.0
Large city	—	31.8
Suburb of large city	—	10.9

Abbreviations: FIES, Food Insecurity Experience Scale; PPP, purchasing power parity.

1 Food insecurity: measured with the 8-item FIES. FAO equates national scales to a Global Standard Scale using a Rasch model and maps country-specific raw-score cut-points. “Moderate or severe” equals 1 when the respondent’s raw score meets or exceeds the mapped threshold, 0 otherwise.

2 Social capital: binary indicator of perceived instrumental support, equal to 1 if the respondent reports having relatives or friends to count on for help, 0 otherwise; aligns with cognitive social capital. Table values reflect the proportion with support.

3 International dollars: household income reported in 27 brackets constructed in PPP-adjusted international dollars; higher brackets indicate higher PPP-adjusted annual household income.

Educational attainment was moderate, with 28.4% of respondents having only a primary education, 51.7% completing secondary school, and 19.8% having earned a college degree. Household income was reported in 27 brackets of annual household income (PPP-adjusted international dollars); the mean bracket index was 13.4 (SD = 6.34), which placed the typical household within the 13th bracket—roughly corresponding to an annual income range of 10,001–12,500 international dollars. Regarding marital status, 56.9% were married or living with a domestic partner, 28.9% were single or never married, 6.67% were separated or divorced, and 7.47% were widowed. About 25% of respondents reported having problems that prevented them from doing activities typical for their age. Respondents’ place of residence was fairly evenly distributed: 23.3% lived in rural areas or on farms, 34.0% resided in small towns or villages, 31.8% in large cities, and 10.9% in suburban areas of large cities.

### Associations and modification at individual level

Social capital modified the association between each individual characteristic and food insecurity. Equally, each individual characteristic modified the association between social capital and food insecurity.

#### Employment

Compared with those without social capital, those with social capital had smaller differences in food insecurity between the fully employed for an employer and each other category of employment. For example, the difference in food insecurity between individuals employed full-time for an employer and individuals unemployed was 0.0611 smaller among those with social capital (Table 2). Similar modifications were seen for other categories of employment. Social capital was associated with 14.26 percentage points lower food insecurity among individuals employed full-time for an employer, 15.69 percentage points lower food insecurity among those who were self-employed full-time, 15.97 percentage points lower among those employed part-time who did not seek full-time work, and 17.40 percentage points lower among those out of the workforce. The magnitude of the association was higher among those facing greater employment insecurity: social capital was associated with 18.67 percentage points lower food insecurity among part-time workers seeking full-time employment and 20.37 percentage points lower among unemployed individuals (Supplementary Figure 1).

**TABLE 2:** Estimated association of social capital with moderate or severe food insecurity by individual characteristics, Gallup World Poll 2014–2021, 115 countries

Individual characteristic	Coefficient for interaction	95% confidence interval for interaction	Association of social capital
Employment status (*n* = 695,239)			
Employed full time for employer (reference)	—	—	−0.1426
Employed full time for self	−0.0143	−0.0211, −0.0075	−0.1569
Employed part time do not want full time	−0.0171	−0.0263, −0.0080	−0.1597
Out of workforce	−0.0314	−0.0370, −0.0259	−0.1740
Employed part time want full time	−0.0441	−0.0523, −0.0358	−0.1867
Unemployed	−0.0611	−0.0696, −0.0526	−0.2037
Educational attainment (*n* = 699,578)			
Primary (reference)	—	—	−0.1696
Secondary	0.0201	0.0157, 0.0245	−0.1495
College	0.0736	0.0665, 0.0806	−0.0960
Income bracket (*n* = 702,533)			
1st bracket (0–365)	—	—	−0.2017
7th bracket (2701–3200)	—	—	−0.1651
13th bracket (10,001–12,500)	—	—	−0.1285
19th bracket (30,001–35,000)	—	—	−0.0919
Marital status (*n* = 694,819)			
Single/never married (reference)	—	—	−0.1573
Married	−0.0077	−0.0127, −0.0027	−0.1650
Domestic partner	−0.0208	−0.0305, −0.0112	−0.1782
Separated	−0.0296	−0.0426, −0.0167	−0.1870
Divorced	−0.0350	−0.0460, −0.0240	−0.1923
Widowed	−0.0395	−0.0477, −0.0313	−0.1968
Physical health (*n* = 681,744)			
Yes (reference)	—	—	−0.1844
No	0.0413	0.0369, 0.0456	−0.1431
Residency (*n* = 700,225)			
Rural (reference)	—	—	−0.1736
A small town or village	0.0017	−0.0035, 0.0070	−0.1718
A suburb of a large city	0.0037	0.0047, 0.0157	−0.1699
A large city	0.0102	−0.0040, 0.0113	−0.1633

Note: observations for all individual characteristics were from 115 countries.

All *P* values for interaction terms were < 0.003 except for residency: A small town or village (*P* = 0.515) and a suburb of a large city (*P* = 0.347). The interaction term for income was 0.0061 with 95% confidence interval (0.0058, 0.0065).

#### Educational attainment

Compared with those without social capital, those with social capital had 0.0201 and 0.0736 smaller differences in food insecurity between primary education and, respectively, secondary and college education. Having social capital was associated with 16.96 percentage points lower food insecurity among individuals with primary education. The estimated association with social capital was lower as educational attainment was higher; among individuals with secondary education, social capital was associated with 14.95 percentage points lower food insecurity, and among those with college education, 9.60 percentage points lower (Supplementary Figure 2).

#### Income

Compared with those without social capital, those with social capital had a 0.0061 smaller association between income and food insecurity. Among individuals at the middle income bracket (13th bracket), social capital was associated with 12.85 percentage points lower food insecurity. Among individuals at the seventh income bracket (∼6 brackets below the mean), social capital was associated with 16.51 percentage points lower food insecurity, whereas among individuals at the 19th income bracket (∼6 brackets above the mean), social capital was associated with 9.19 percentage points lower food insecurity (Supplementary Figure 3).

#### Marital status

Compared with those without social capital, those with social capital had smaller differences in food insecurity between being single/never married and each other category of marital status. Social capital was associated with 15.73 percentage points lower food insecurity among individuals who were single. The estimated association between social capital and food insecurity differed by marital status, with 16.50 percentage points lower food insecurity among those who were married, 17.82 percentage points lower among those living with a domestic partner, 18.70 percentage points lower among those who were separated, 19.23 percentage points lower among those who were divorced, and 19.68 percentage points lower among individuals who were widowed (Supplementary Figure 4).

#### Physical health

Compared with those without social capital, those with social capital had a 0.0413 smaller difference in food insecurity between those with and without health problems. Social capital was associated with 14.31 percentage points lower food insecurity among individuals who reported that health problems did not limit their ability to engage in activities typical for their age. Among individuals who reported that health problems did limit their activities, social capital was associated with 18.44 percentage points lower food insecurity (Supplementary Figure 5).

#### Residency

Compared with those without social capital, those with social capital had smaller differences in food insecurity between residents in rural areas and residents in each other category of residency. Social capital was associated with 17.36 percentage points lower food insecurity among individuals who resided in a rural area, 16.33 percentage points lower food insecurity among individuals who resided in a large city, 16.99 percentage points lower food insecurity among those who resided in a suburb of a large city, and 17.18 percentage points lower food insecurity among those in a small town or village (Supplementary Figure 6).

### Among-country variability in the association of social capital

A random coefficient model was used to account for variability in the association between social capital and moderate or severe food insecurity across 115 countries. Overall (across all countries), having social capital was negatively associated (*β* = −0.1647) with the probability of experiencing moderate or severe food insecurity. The strength of this association varied substantially among countries (SD = 0.0570). The country-specific slopes ranged from −0.3180 to −0.0549, indicating that although the slope remained negative in all countries, the magnitude of the country-specific slopes varied considerably around the overall slope.

**FIGURE 2:**
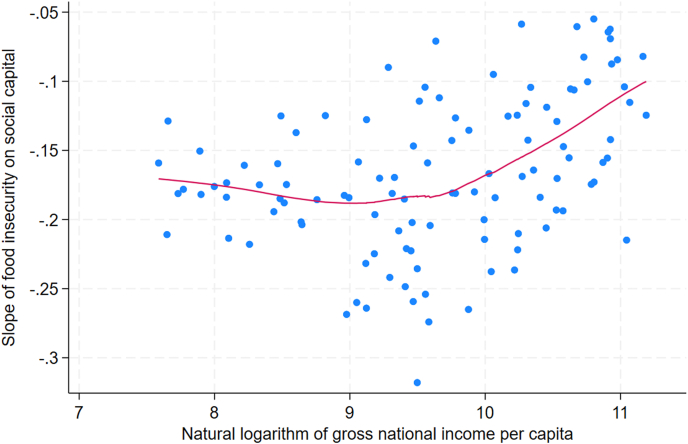
Locally weighted regression of the country slope of food insecurity on social capital vs. log GNI per capita, Gallup World Poll 2014–2021, 115 countries (regression sample *n* = 112). Note: approximate mappings of the log scale: In 7∼$1.1k, In In 8 ∼ In In 9∼ $8.1k, In 10 ∼ $22.Ok, In 11 ∼ $59.9k.

A locally weighted regression plot showed that the country-specific slope for food insecurity on social capital was relatively flat across low- and middle-income countries but rose with log-GNI in higher-income countries (Figure 2). The inflection point in the plot occurred near log-GNI of 10, corresponding to ∼22,000 international dollars. A quadratic model regressing the slope on log-GNI confirmed a curvilinear relationship with a positive quadratic coefficient and improved fit over a linear model, explaining 24.48% of the variation in the country-specific slopes compared with 12.93% for the linear model (Table 3). A model that had added a cubic term explained 23.82% of the variation.

**TABLE 3:** Coefficients from regression of country-specific social capital-food insecurity association on national income, Gallup World Poll 2014–2021, 115 countries (regression sample *n* = 112)

	Linear model				Quadratic model		
	Coefficient	95% confidence interval		*P* value	Coefficient	95% confidence interval	*P* value
Log-GNI per capita	0.0210	0.0111, 0.0310		<0.001	−0.3595	−0.5384, −0.1806	<0.001
log-GNI^2^	—	—		—	0.0201	0.0107, 0.0295	<0.001
Constant	−0.3693	−0.4658, −0.2728		<0.001	1.4139	0.5718, 2.2560	<0.001

Note: outcome is the country-specific slope estimate for the association between social capital and moderate or severe food insecurity. Models used ordinary least squares regressions with mean national GNI per capita (2014–2021), log-transformed, as the predictor. The linear and quadratic models explained 12.93% and 24.48% of the variance in the country-specific slopes, respectively. A model that had added a cubic term (not shown) explained 23.82% of the variation.

Abbreviation: GNI, gross national income.

## Discussion

The association between cognitive social capital and moderate or severe food insecurity was stronger in magnitude among individuals in strata associated with a higher probability of food insecurity. Greater social capital was associated with a lower probability of moderate or severe food insecurity, with attenuation of this association in higher-income contexts.

Models with interaction terms for individual characteristics indicated buffering. The association between cognitive social capital and food insecurity was stronger in magnitude among individuals with primary education, lower income, unemployment, limited physical health, and disrupted marital ties. These patterns are consistent with the stress model, in which social resources act through compensation that lowers risk and buffering that reduces the effect of high stress by shaping appraisal, coping, and behavioral responses [10,11,32].

The interaction pattern aligns with Thoits’s account of stress buffering [32]. Routine ties maintain self-esteem, mattering, and perceived control in noncrisis periods, which lowers threat appraisal when stress rises; under major stress, support becomes visible and targeted, altering appraisal and dampening emotional and physiological responses. The key mediators are cognitive (perceived support availability, belonging, mattering, self-esteem, and sense of control), so effects are expected to be largest at high stress [32]. This mechanism maps onto the observed modifiers: vulnerability elevates exposure and appraised threat, making cognitively mediated support more consequential in low income, unemployment, poor health, and disrupted marital ties, where the associations were largest in magnitude.

Evidence from high-stress settings is consistent with this interpretation. Among mothers affected by Peru’s 2017 coastal flooding, severe food insecurity was 90.0% at ∼3 mo post disaster and 31.8% by ∼8 mo; over the same period, cognitive social capital increased from 42.1% to 86.7% [33]. Early support came from proximate sources (e.g., relatives, neighbors, religious groups, and local authorities), and the perceived ability to count on others remained high during recovery. These co-occurring shifts are consistent with buffering, in which cognitive social capital operates most strongly under acute strain.

Country-level results showed an association between social capital and food insecurity in each country, with substantial among-country variation and nonlinear attenuation at higher national income. This pattern accords with Lin’s network theory: social capital comprises embedded resources that actors access and mobilize for instrumental returns; when public or market provision is limited, networked access compensates, whereas as institutional access expands with income, formal systems substitute for personal ties and the marginal return to social capital declines [34]. Variation in country-specific slopes is also consistent with Lin’s strength-of-position proposition: returns depend on locations in status and authority hierarchies and on network configuration. Settings with more bridging and linking ties, and greater mobilization capacity, are expected to translate embedded resources into food security more effectively; settings dominated by bonding ties alone may yield weaker associations despite greater need. The observed inflection near log-GNI of 10 (∼22,000 international dollars) aligns with diminishing marginal returns as institutional capacity rises.

These findings extend Smith et al. [12], who reported negative associations across economic contexts but did not examine heterogeneity. Although social capital was associated with food insecurity in all countries, the magnitude of association was lower with higher national income. This variation likely reflects how effectively social capital is mobilized, given available resources [[34], [35], [36]]. In lower-income settings, personal networks may substitute for absent provision. In higher-income settings, linking and bridging ties may function more as connectors to services than as sources of direct material aid, yielding smaller marginal effects even when the association remains, albeit attenuated.

Distribution and functioning of social capital can further explain differences among countries. In India, rising inequality weakened bridging ties and reinforced bonding ties, limiting collective benefits [37]. In Turkey, politically concentrated linking ties produced uneven conversion of social ties into instrumental returns, amplifying benefits for some individuals while limiting benefits for others, which can reduce slope magnitude even when the direction is conserved [13].

Social capital operates through 2 complementary processes at different scales: buffering and compensation. At the individual level, perceived availability of functional support buffers the impact of stress on well-being [10]. This buffering operates through appraisal and coping: esteem support offsets threats to self-worth and informational support facilitates reappraisal and selection of coping responses. Buffering is strongest when support matches the stressor. Evidence indicates that specific functional measures (e.g., having a confidant) more reliably detect stress–support interactions than global structural measures [10,11]. Our measure of cognitive social capital, having relatives or friends to count on, accords with this functional path.

At the macro level, social capital functions as a structural resource that compensates for risk by improving overall conditions, independent of acute stress exposure, through integration, predictability, and role stability [34]. Lin’s network theory specifies how such structural effects arise: social capital consists of resources embedded in networks that can be accessed and mobilized to yield returns via information, influence over gatekeepers, social credentials, and reinforcement. Returns depend on network locations, hierarchical positions, and the stock and distribution of embedded resources [34]. Structural and positional variation, shaped by institutions and development, produces inequality in accessible and mobilized social capital and, therefore, heterogeneity in country-level outcomes across countries.

Buffering and compensation can be viewed as scale-specific aspects of the same network-resource process. At the micro level, individuals mobilize functional support within reachable ties to change threat appraisal (buffering) [34]. At the macro level, societies differ in the distribution and mobilization of embedded resources: open networks with bridging ties to higher-status contacts enhance instrumental returns, whereas dense bonding networks preferentially preserve expressive returns; institutional arrangements shift the balance between density and openness and thereby the magnitude of structural compensation [34].

The country-level findings are consistent with compensation and institutional substitution. Integration and positional reach are associated with a lower probability of food insecurity [34]. The marginal return to social capital attenuates as national income rises, consistent with formal systems substituting for personal ties. Where institutional supply is constrained, networked access compensates for missing public or market provision and the association is stronger; where institutional access is broader, the association remains but is weaker in magnitude because needs are partly met through formal channels, like cash transfer programs.

Illustrative program evidence is consistent with this dual-mechanism account. In Malawi, training that organized village savings and loans groups increased within-group trust and cooperation, whereas a cash lump sum alone did not, consistent with cognitively mediated buffering activated through proximate ties, mutual aid, and flexible coordination under strain [38]. At the same time, effects depended on structural conditions, including program reliability, shock exposure, and targeting legitimacy. Several groups collapsed with floods or drought and with suspensions of social cash transfer payments, whereas no closures occurred in better-off clusters facing the same shocks; jealousy and perceived exclusion around targeting reduced vertical and out-group trust. These features mirror the country attenuation observed: where formal systems have limited coverage, networks are most needed yet fragile; where resources have broad coverage, cohesion is more durable, but marginal returns to social capital are smaller because formal provision substitutes for aid delivered through personal and community ties.

Eight GWP waves (2014–2021) across 115 countries enabled globally comparable estimation with the FAO-validated FIES [19]. Social capital was operationalized with a single binary item (“having relatives or friends to count on”) [4], maximizing cross-national comparability and field efficiency but capturing only 1 cognitive component; structural components were not analyzed. Fixed-effects models controlled for time-invariant country-level factors and year. Random-coefficient models quantified variability among countries in the magnitude of association. The resulting estimates remain associational because the analyses were cross-sectional; unmeasured confounding cannot be ruled out. Standardized survey procedures promoted measurement consistency across countries, with acknowledgment that mode- or context-related variance may persist and warrants consideration in interpretation.

The association between cognitive social capital and food insecurity was not uniform: the magnitude of association was larger in strata associated with a higher probability of food insecurity and was attenuated in higher-income national contexts, consistent with partial substitution between personal ties and formal systems. At the individual level, the larger magnitude of association in strata with a higher probability of food insecurity is consistent with buffering, in which perceived access to functional support is more strongly linked to food insecurity under greater constraints. At the country level, the nonlinear attenuation of country-specific associations with higher national income is consistent with partial institutional substitution, such that the marginal association of personal ties is smaller where formal systems and markets meet a greater share of needs. These results suggest that cognitive social capital is more strongly linked to food insecurity in low-resource strata and lower-income settings, and less strongly linked in contexts with broader institutional access.

The results support a context-dependent interpretation in which cognitive social capital operates alongside, rather than replaces, material and institutional supports. These analyses do not provide causal evidence that strengthening social capital will reduce food insecurity; rather, they identify where cognitive social capital is most strongly linked to food insecurity and where formal provisions may partially substitute for informal support. Under greater structural constraints, informal support is more strongly linked to food insecurity because fewer formal and material options are available.

Cognitive social capital was more strongly linked to food insecurity under greater individual and institutional constraints, and less strongly linked where formal systems meet a larger share of needs. This interpretation highlights the importance of modeling and reporting heterogeneity across social and institutional contexts, and it provides a basis for specifying effect modification in future research rather than interpreting a single pooled estimate as universally applicable. For program design and evaluation, stratified reporting and tests of effect modification by socioeconomic and health strata within countries and by national institutional context across countries are warranted so that targeting and impact assessment are not based on a single pooled estimate. In practice, such results can inform context-specific refinement of program delivery and governance features (e.g., accessibility and coordination with existing community support structures) in settings where formal provision is constrained and informal support plays a larger role, while recognizing that such adjustments require causal and implementation evidence to establish effectiveness. For food systems governance, the observed cross-national attenuation implies that institutional arrangements and coverage shape how strongly informal support networks are implicated in food insecurity, which is relevant for interpreting the distribution of food security programs across settings and for harmonizing expectations in cross-border funding and monitoring. Future research that clarifies practical implications includes designs to test causality; implementation studies that examine whether and how program features influence cognitive and structural components of social capital; and measurement work that distinguishes bonding, bridging, and linking social capital to identify which forms, and in which institutional contexts, are most strongly associated with a lower probability of food insecurity. Such work can clarify when social capital complements, rather than substitutes for, formal provision in reducing food insecurity. Cognitive social capital should be understood not as a universal substitute for material and institutional support, but as a context-sensitive resource whose relevance to food insecurity depends on individual constraints and institutional capacity.

## Author contributions

The authors’ responsibilities were as follows – EAF: designed research; KO’C: provided essential materials; KO’C, SI, EAF: analyzed data; SI, EAF: had responsibility for revisions and final content; and all authors: drafted the manuscript and read and approved the final manuscript.

## Data availability

Gallup, Inc., and the Voices of the Hungry project, FAO, provided access to the Gallup World Poll and Food Insecurity Experience Scale data.

## Funding

This research was supported in part by a Cooperative Research Agreement (agreement 58-4000-2-0055) from the US Department of Agriculture, Economic Research Service.

## Conflict of interest

The authors report no conflicts of interest. The findings and conclusions in this presentation are those of the authors and should not be construed to represent any official USDA or U.S. Government determination or policy.
