# Supplementary material for: Individual Characteristics and National Income Modify the Association between Cognitive Social Capital and Food Insecurity: Evidence from the Gallup World Poll, 2014–2021
Source: Curr Dev Nutr. 2026 Feb 6;10(3):107654. doi: 10.1016/j.cdnut.2026.107654 (PMC12966665; doi:10.1016/j.cdnut.2026.107654)

Supplementary Material for: Individual characteristics and national income modify the association between cognitive social capital and food insecurity: Evidence from the Gallup World Poll, 2014–2021

Sejla Isanovic[1]*, Kegan O'Connor[2], Audrey L. Richards[1], Edward A. Frongillo[1]

[1]Department of Health Promotion, Education, and Behavior, Arnold School of Public Health, University of South Carolina, Columbia, SC, USA

[2]United States Department of Agriculture, Economic Research Service, Washington, DC, USA

Footnotes:

The findings and conclusions in this presentation are those of the authors and should not be construed to represent any official USDA or U.S. Government determination or policy.

This research was supported in part by a Cooperative Research Agreement (agreement 58-4000-2-0055) from the U.S. Department of Agriculture, Economic Research Service.

***Correspondence:** Sejla Isanovic, sejla@email.sc.edu

Individual characteristics and national income modify the association between cognitive social capital and food insecurity: Evidence from the Gallup World Poll, 2014–2021
Sejla Isanovic

Supplementary Table 1. Descriptive information and data availability for variables used and not used in the analysis.

Variable	Role	Description / Item wording	Source	Response options	Frequency	Total N (all response categories)	Non-missing substantive responses (n)	Substantive response coverage (%)	Don't know (n)	Refused (n)
Education	Effect modifier	Highest education attained.	Gallup World Poll (item: WP3117)	Elementary education or less	233,496	824,738	820,837	99.5%	1911	1990
				Secondary–3 year Tertiary Secondary (9-15 years of education)	424,461					
				Completed 4 years of education beyond 'high school' and/or received 4-year college degree	162,880					
				Don't know	1,911					
				Refused	1,990					
Employment	Effect modifier	Employment status.	Gallup World Poll	Employed full time for an employer	237,311	817,254	817,254	100.0%	0	0
				Employed full time for self	112,706					
				Employed part time do not want full time	56,340					
				Unemployed	50,537					
				Employed part time want full time	63,000					
				Out of workforce	297,360					
Income bracket index	Effect modifier	Worldwide household income brackets (international dollars).	Gallup World Poll	0–365	32,413	858,641	858,641	100.0%	0	0
				366–730	31,432					
				731–1200	38,656					
				1201–1700	24,572					
				1701–2200	11,683					
				2201–2700	37,984					
				2701–3200	31,754					
				3201–4000	22,953					
				4001–5000	18,852					
				5001–6000	37,648					

Variable	Role	Description / Item wording	Source	Response options	Frequency	Total N (all response categories)	Non-missing substantive responses (n)	Substantive response coverage (%)	Don't know (n)	Refused (n)
				6001–7500	48,208					
				7501–10,000	18,830					
				10,001–12,500	20,984					
				12,501–15,000	51,095					
				15,001–17,500	39,841					
				17,501–20,000	9,032					
				20,001–25,000	18,932					
				25,001–30,000	72,772					
				30,001–35,000	29,777					
				35,001–40,000	4,943					
				40,001–50,000	22,426					
				50,001–60,000	56,058					
				60,001–75,000	23,122					
				75,001–100,000	15,698					
				100,001–125,000	23,131					
				125,001–150,000	47,557					
				≥150,001	34,144					
Marital status	Effect modifier	What is your current marital status?	Gallup World Poll (item: WP1223)	Single/Never been married	236,176	821,261	816,405	99.4%	3158	1698
				Married	412,813					
				Separated	20,126					
				Divorced	34,325					
				Widowed	60,971					
				Domestic partner	51,994					
				Don't know	3,158					
				Refused	1,698					
Physical health	Effect modifier	Do you have any health problems that prevent you from doing any of the things people your age normally can do?	Gallup World Poll (item: WP23)	Yes	186,377	754,973	751,736	99.6%	2705	532
				No	565,359					
				Don't know	2,705					
				Refused	532					
Residency		Residency category.		Rural area or on farm	191,628	824,761	821,862	99.6%	2307	592

Variable	Role	Description / Item wording	Source	Response options	Frequency	Total N (all response categories)	Non-missing substantive responses (n)	Substantive response coverage (%)	Don't know (n)	Refused (n)
	Effect modifier		Gallup World Poll (item: WP14)	Small town or village<br>Large city<br>Suburb of large city<br>Don't know<br>Refused	279,777<br>261,088<br>89,369<br>2,307<br>592					
Education access	Effect modifier (not used)	Feels education is accessible to anyone, regardless of economic situation.	Gallup World Poll (item: WP843); Smith et al., 2017	Yes<br>No<br>Don't know<br>Refused	39,796<br>23,792<br>1,401<br>159	65,148	63,588	97.6%	1401	159
Employment hours worked	Effect modifier (not used)	Hours worked per week.	Frongillo et al., 2017	Less than 15<br>15 to 29<br>30 to 39<br>40 to 49<br>50 or more<br>No answer	36,696<br>40,850<br>56,715<br>128,517<br>88,678<br>8,402	359,858	351,456	97.7%	0	0
Household size	Effect modifier (not used)	How many children under 15 years of age are now living in your household?	Gallup World Poll (item: WP1230); Frongillo et al., 2017	Number children under 15 in household	823,760	823,760	823,760	100.0%	0	0
Household size	Effect modifier (not used)	Including yourself, how many people, age 15 or older, currently live in this household?	Gallup World Poll (item: WP12); Frongillo et al., 2017	Residents 15+ in Household	823,757	823,757	823,757	100.0%	0	0
Urbanicity	Effect modifier (not used)	Rural population (percentage of total) Calculated as the difference between total population and urban population.	World Bank	% rural of total population	812,668	812,668	812,668	100.0%	0	0

Variable	Role	Description / Item wording	Source	Response options	Frequency	Total N (all response categories)	Non-missing substantive responses (n)	Substantive response coverage (%)	Don't know (n)	Refused (n)
Urbanicity	Effect modifier (not used)	Urban population (percentage of total) Calculated using World Bank population estimates and urban ratios from the United Nations World Urbanization Prospects.	World Bank	% urban of total population	812,668	812,668	812,668	100.0%	0	0
Social capital (cognitive)	Exposure	If you were in trouble, do you have relatives or friends you can count on to help you whenever you need them, or not?	Gallup World Poll (item: WP27)	Yes	576,429	709,628	702,850	99.0%	6260	518
				No	126,421					
				Don't know	6,260					
				Refused	518					
Social life index	Exposure (not used)	Feels can count on friends and family in times of need (WP27) and Satisfied with ability to make friends (WP10248).	Gallup World Poll	0	38,926	613,933	613,933	100.0%	0	0
				50	176,900					
				100	398,107					
Social network	Exposure (not used)	In the city or area where you live, are you satisfied or dissatisfied with the opportunities to meet people and make friends?	Gallup World Poll (item: WP10248); Smith et al., 2017	Satisfied	475,440	613,933	590,125	96.1%	22237	1571
				Dissatisfied	114,685					
				Don't know	22,237					
				Refused	1,571					
Social network	Exposure (not used)	Someone in life always encourages to be healthy.	Gallup World Poll (item: WP14444)	Strongly disagree	10,614	199,025	197,525	99.2%	1259	241
				Disagree	13,120					
				Neutral	33,416					
				Agree	55,236					
				Strongly agree	85,139					
				Don't know	1,259					

Variable	Role	Description / Item wording	Source	Response options	Frequency	Total N (all response categories)	Non-missing substantive responses (n)	Substantive response coverage (%)	Don't know (n)	Refused (n)
				Refused	241					
Social network	Exposure (not used)	Friends and family give positive energy every day.	Gallup World Poll (item: WP14445)	Strongly disagree	9,250	199,025	197,609	99.3%	1199	217
				Disagree	12,685					
				Neutral	35,506					
				Agree	56,921					
				Strongly agree	83,247					
				Don't know	1,199					
				Refused	217					
Food insecurity	Outcome	Individual experienced moderate or severe food insecurity within the last 12 months. Proportion of people in population represented by sampled person whose food insecurity exceeds set threshold "ate less than they thought they should eat" on Voice of Hunger global standard scale.	Gallup World Poll; Frongillo et al., 2017; Smith et al., 2017	Yes	824,598	824,598	824,598	100.0%	0	0
Food insecurity	Outcome (not used)	Individual experienced most severe food insecurity within the last 12 months.	Smith et al., 2017	Yes	824,598	824,598	824,598	100.0%	0	0
Food insecurity	Outcome (not used)	Have there been times in the past 12 months when you or other members of your household went without Enough food to eat?	Gallup World Poll (item: WP40)	Yes	269,283	822,759	818,737	99.5%	3195	827
				No	549,454					
				Don't know	3,195					
				Refused	827					

Individual characteristics and national income modify the association between cognitive social capital and food insecurity: Evidence from the Gallup World Poll, 2014–2021

Sejla Isanovic

Supplementary Figures 1–6 are post-estimation plots of the estimated associations between cognitive social capital and moderate or severe food insecurity, stratified by individual characteristics.

Supplementary Figure 1. Estimated association of social capital with moderate or severe food insecurity by employment status

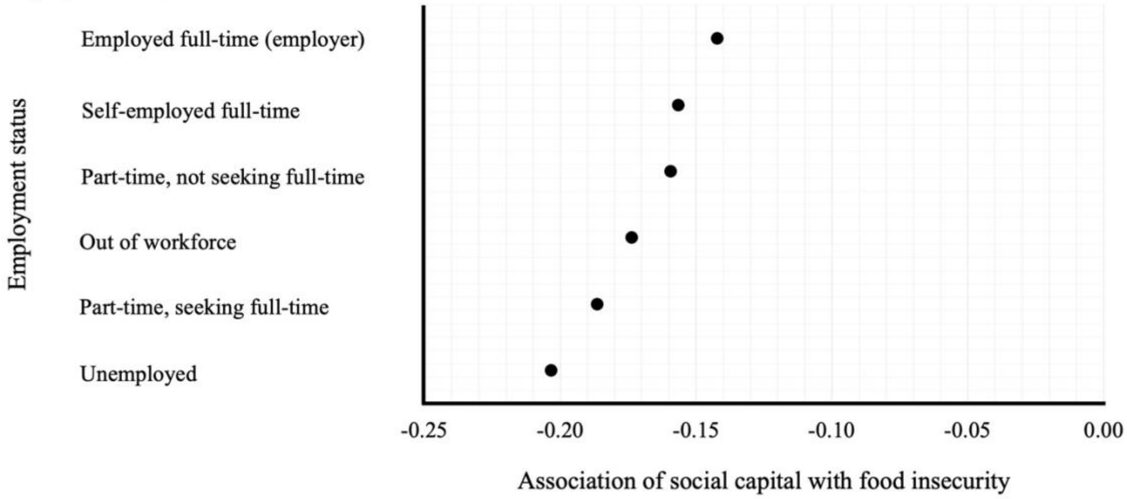

Supplementary Figure 2. Estimated association of social capital with moderate or severe food insecurity by educational attainment

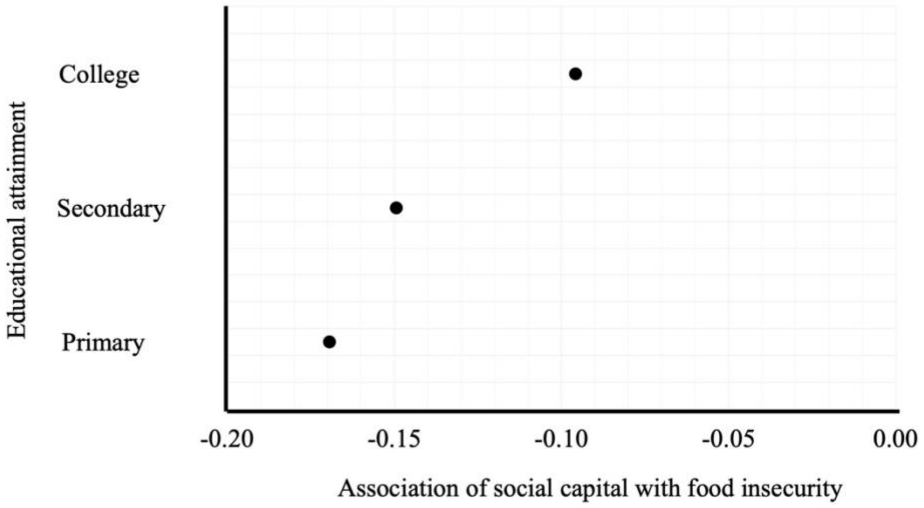

Supplementary Figure 3. Estimated association of social capital with moderate or severe food insecurity by household income

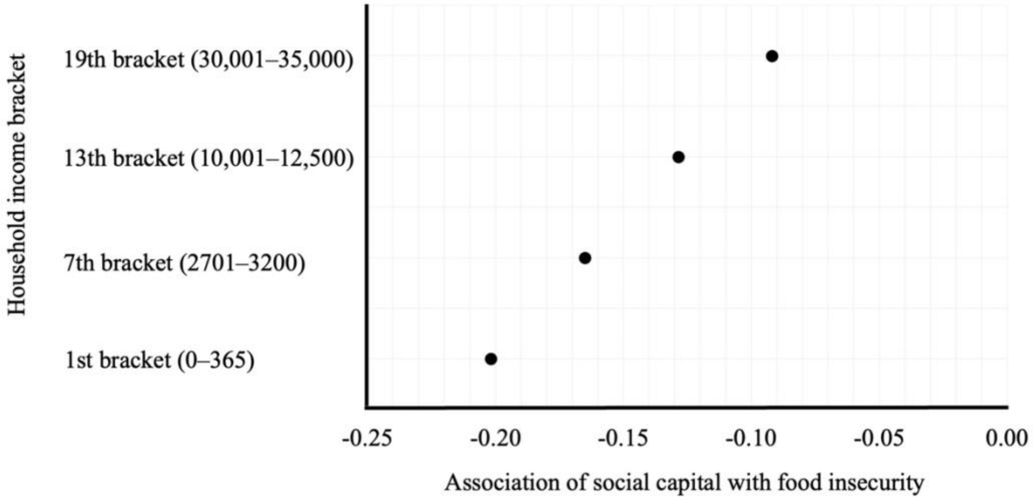

Supplementary Figure 4. Estimated association of social capital with moderate or severe food insecurity by marital status

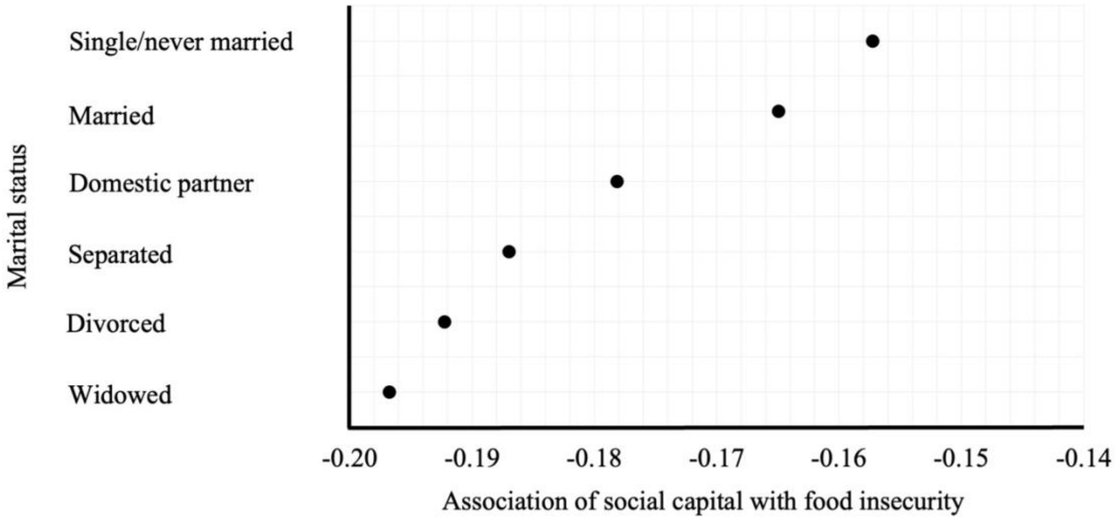

Supplementary Figure 5. Estimated association of social capital with moderate or severe food insecurity by physical health

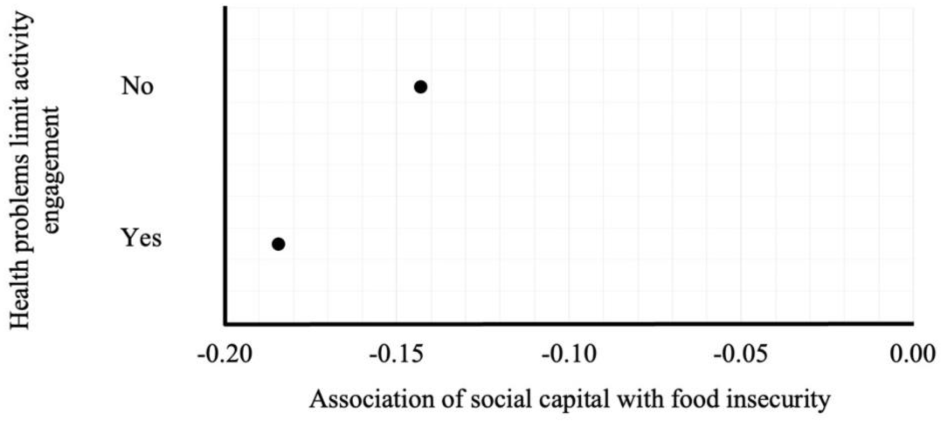

Supplementary Figure 6. Estimated association of social capital with moderate or severe food insecurity by residency

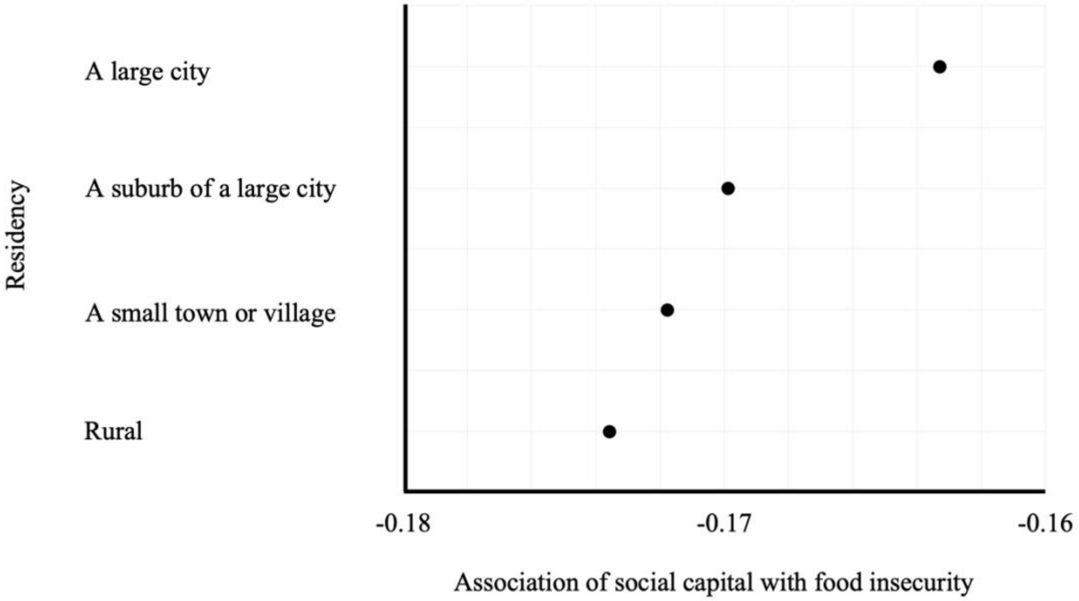

Supplement: Multimedia component 1 [file mmc1.pdf]
